# Impact of mother's mass media exposure on early childhood development in pakistan: urban vs. rural analysis

**DOI:** 10.1186/s12889-025-24586-z

**Published:** 2025-11-03

**Authors:** Muhammad Afiq, Mahniya Zafar

**Affiliations:** 1 The Urban Unit, Planning & Development Board (P&D), Government of the Punjab, Lahore, Pakistan; 2 Centre for Research in Economics and Business (CREB), Lahore School of Economics, Office: Room 10, Lahore, Pakistan

## Abstract

**Background:**

Mothers play a pivotal role in early childhood development (ECD), and mass media serves as a key channel for shaping maternal knowledge and practices.

**Objective:**

This study examines how maternal exposure to five types of mass media—newspapers, radio, television, internet, and mobile phones—affects ECD outcomes in Pakistan, with a focus on rural-urban disparities.

**Methods:**

We used nationally representative data from the 2014 and 2018 waves of Pakistan’s Multiple Indicator Cluster Survey (MICS), analyzing 73,866 children aged 3–4 years. ECD was assessed across four domains: physical health, literacy-numeracy, learning, and social-emotional development. Poisson regression with robust variance was employed to assess associations between media exposure (type and frequency) and ECD outcomes, adjusting for maternal and household characteristics.

**Results:**

Exposure to a greater variety of media types was positively associated with improved ECD, with each additional media type linked to a 5% improvement in rural areas and 8.15% in urban areas. However, excessive media frequency had a marginally negative effect. Among all media types, mobile phone use had the strongest impact in rural settings. Other key predictors of ECD included early education, access to books, and parental stimulation, while malnutrition negatively affected outcomes. Regional disparities were observed, with Punjab outperforming other provinces and Balochistan showing the lowest ECD indicators.

**Conclusion:**

This study highlights the potential of media-based interventions to empower mothers and improve child development outcomes. Tailored campaigns using accessible and frequently used media can bridge knowledge gaps, promote equitable development, and address persistent regional inequalities.

## Introduction

Early childhood development (ECD) is crucial for shaping a child’s social, cognitive, and physical growth, with lifelong impacts on health and success [1, 2]. Brain development peaks in the first five years, making interventions during this period the most effective [3]. Yet, nearly 200 million children globally face malnutrition, while 43% under five are at risk of developmental delays due to poverty, hunger, and limited access to basic services [4, 5]. These challenges highlight the urgency of early action.

ECD is central to the UN’s Sustainable Development Goals (SDG 4), which emphasize inclusive education [5, 6]. Target 4.2.1 specifically focuses on early education access, recognizing its role in lifelong learning and well-being. Despite this, millions in developing countries fail to meet cognitive, social, or motor milestones—over 200 million under five in low- and middle-income nations alone [7]. Delays are linked to factors like parental education, household wealth, and access to early education, stressing the need for holistic, equity-driven solutions [8, 9].

Early Childhood Development (ECD) encompasses four critical domains: cognitive, physical, social-emotional, and language development, each shaping a child’s ability to thrive [10]. Cognitive development involves problem-solving, memory, and decision-making skills, while physical development focuses on motor skills and healthy growth, often impacted by nutrition [11]. Social-emotional development is essential for forming relationships and emotional regulation, supported by responsive caregiving environments [12]. Language development, crucial for communication, is fostered through early and meaningful interactions with caregivers [13]. A comprehensive approach addressing all four domains can mitigate developmental delays and promote lifelong well-being.

While media is widely accessible, its role as a *behavioral change agent* in ECD remains underexplored—especially in contexts like Pakistan. Most existing studies overlook how indirect information exposure, such as through media, can shape parenting behaviors and, by extension, child development outcomes [14]. Media is widely accessible, but its influence on parenting practices and children’s development, particularly across rural and urban areas, remains unclear. Media platforms such as television, radio, mobile phones, and newspapers play a crucial role in sharing parenting advice, promoting health practices, and supporting educational strategies, particularly in resource-constrained environments [15]. This study aims to explore how mothers’ exposure to these five media types affects ECD outcomes in both rural and urban regions of Pakistan.

Traditional interventions focus on health and education, emerging research highlights mass media’s potential to “transform” parenting practices [16]. In Pakistan, where formal education access is uneven, media bridges gaps by disseminating child-rearing knowledge [17]. Mobile phones, for instance, empower mothers in low-resource areas with health/nutrition information via apps or SMS [18, 19], correlating with better healthcare access [20].

Resources like health care, education, and media, are mainly divided along urban and rural lines that also mediate access to them. Better knowledge about child development can be expected in urban populations where people have better access to healthcare facilities, educational institution and variety of media [21]. The authors suggest that exposure to media in rural settings has a larger impact on improving parenting behaviors than in the urban settings where they are more accessible. As a result, marketing campaigns aimed at rural populations should gain more benefits in positive effect on child development [8, 22].

There are clear differences between rural and urban areas in access to resources and development opportunities. The rural people had less access to healthcare specialists, blogs, magazines, and search engines compared to those in urban areas [23]. This gap was worse for those with low health literacy. However, these studies often fail to disentangle whether increased access to media in rural areas *compensates* for other disadvantages—a critical question we explore by comparing effect sizes across rural and urban populations [24]. Rural people also used the Internet less for health information but relied on other sources like TV and healthcare professionals just as much. These differences were mainly linked to lower education and income levels in rural areas. A study found out that urban environments in Mexico provided benefits, reducing problems in children’s social and cognitive development [10]. These studies suggest that differences between rural and urban areas often depend on a mix of social, economic, and environmental factors.

A child’s age and gender are important factors in early childhood development (ECD) outcomes. Younger children are more likely to show rapid cognitive and physical development, while age-specific interventions can better support their growth. Boys and girls may also experience different developmental trajectories due to social and cultural influences [25]. Salt iodization is another key factor, as iodine deficiency can impair cognitive development, particularly in young children [26, 27]. The presence of books in the household is linked to better cognitive outcomes, as it promotes early literacy and intellectual engagement [27]. Malnutrition negatively affects a child’s development, leading to delays in cognitive, emotional, and physical growth [11, 28]. Access to early childhood education (ECE) is crucial for enhancing ECD outcomes, as it provides structured learning environments that foster social, cognitive, and emotional skills, especially for children from disadvantaged backgrounds [29].

Mothers’ education plays a vital role in shaping early childhood development (ECD) outcomes. Educated mothers are more likely to engage in activities that stimulate cognitive, emotional, and social growth in young children, such as reading, storytelling, or playing educational games [30]. Parental stimulation activities, whether initiated by the mother or father, provide essential learning opportunities that foster language skills and creativity [31, 32]. Additionally, parental age can influence ECD outcomes. Younger parents may bring energy and adaptability to child-rearing, while older parents often have greater experience and stability [13, 33, 34]. A balanced approach that combines parental involvement, educational exposure, and nurturing activities contributes positively to a child’s early development.

Mother parity, or the number of children born to a mother, has been shown to influence early childhood development outcomes. Higher parity is often associated with reduced resources and attention per child, which may negatively affect developmental outcomes [14]. Household head education is often used as a proxy for father’s education, as it reflects the level of parental investment in children’s well-being. A more educated household head tends to provide better resources and support for children’s development [35, 36]. Some studies show that larger households may positively impact early childhood development (ECD) due to more social interactions. However, other studies suggest a negative effect, as larger families may face resource shortages [9, 37, 38]. Wealth quintiles also play a key role in ECD outcomes. Families in higher wealth groups typically have better access to healthcare, education, and nutrition, leading to better development outcomes for children [39].

Despite the growing body of literature on early childhood development, most existing studies have focused primarily on direct interventions such as early education programs or nutrition, with limited attention to the indirect role of parental media exposure—especially maternal exposure—as a determinant of ECD outcomes. Moreover, few studies critically examine the intersection of media exposure and rural-urban disparities, particularly in low- and middle-income countries like Pakistan. This study addresses these gaps by exploring how mothers’ exposure to five types of media influences ECD outcomes, while also analyzing differential effects across rural and urban contexts. By doing so, it contributes novel insights into the indirect pathways through which maternal information environments shape child development. A summary of key literature reviewed, including study context, methods, and findings, is provided in Appendix.

## Methodology

The Multiple Indicator Cluster Surveys (MICS)^1^ were conducted across various provinces and regions of Pakistan in two waves: MICS-5 and MICS-6. MICS5, carried out between 2014 and 2017, covered Punjab, Sindh, Khyber Pakhtunkhwa (KPK), and Gilgit-Baltistan, while MICS6, conducted between 2017 and 2020, included Punjab, Sindh, KPK, and Balochistan. These surveys provide detailed, region-specific, and nationally representative data, enabling an in-depth analysis of early childhood development (ECD) across diverse socioeconomic and geographical contexts. For simplicity, we refer to these survey waves as 2014 (MICS5) and 2018 (MICS6) throughout this study.

The sample for this study consists of children aged three to four years from both survey waves. The 2014 dataset included 11,256 children in Punjab [40], 6,701 in Sindh [41], 8,228 in KPK [42], and 2,660 in Gilgit-Baltistan [43], totaling 28,845 children. The 2018 dataset included 15,727 children in Punjab [44], 7,716 in Sindh [45], 11,630 in Balochistan [46], and 9,948 in KPK [47], totaling 45,021 children. Combining both waves, the dataset initially comprised 73,866 children aged 36–59 months. After removing missing observations, comprehensive information for 71,968 children was used in the analysis.

The study’s primary outcome variable is the Early Childhood Development Index (ECDI), developed using the United Nations Children’s Fund (UNICEF) methodology. The ECDI evaluates children’s physical, cognitive, literacy-numeracy, and socio-emotional development based on ten carefully designed indicators for children aged 36–59 months. Independent variables included maternal and paternal education, household wealth quintile (used as a proxy for socioeconomic status), area of residence (rural/urban), region of residence (province), child’s gender, disability status, and disciplinary practices used by household adults. These variables were examined to assess their associations with ECD outcomes.

### Outcome variable: ECDI

In this study, the outcome variable is the Early Childhood Development Index (ECDI), which indicates whether or not a child is on track developmentally in 4 key domains as shown in Table 1.The ECDI is composed of 10 components, across these four domains, and child is considered ‘on track’ if they meet criteria in at least three of these domains [26, 31]. The mothers of children under five answered questions related to these domains. The ECDI is considered an accurate measure for developmental tracking of children in low- and middle-income countries [8].

**Table 1:** Questions across the four domains of ECD were used, along with a psychometric approach, to determine whether children were developmentally on track

Four domains	10 Questions	Question item on track if the answer is …	On track if
Literacy-numeracy	1. Can the child identify or name at least ten letters of the alphabet?	Yes	At least two items on track
	2. Can the child read at least four simple, popular words?	Yes	
	3. Does the child know the name and recognize the symbol of all numbers from 1 to 10?	Yes	
Physical	4. Can the child pick up a small object with two fingers, like a stick or a rock from the ground?	Yes	At least one items on track
	5. Is the child sometimes too sick to play?	No	
Learning	6. Does the child follow simple directions on how to do something correctly?	Yes	At least one items on track
	7. When given something to do, is the child able to do it independently?	Yes	
Social-emotional	8. Does the child get along well with other children?	Yes	At least two items on track
	9. Does the child kick, bite, or hit other children or adults?	No	
	10. Does the child get distracted easily?	No	

### Mother media use (main independent variable)

The key exposure variable in this study was maternal use of mass media, which included newspapers or magazines, radio, television, the internet, and mobile phones. Mothers were asked about their frequency of engaging with each type of media. For newspapers, magazines, radio, and television, participants were asked how often they read, listened to, or watched these media types, with response options including: not at all, less than once a week, at least once a week, and almost every day.

For internet usage, the 2014 survey (MICS5) asked participants how frequently they had used the internet during the past one month, while the 2018 survey (MICS6) extended the timeframe to the past three months. Both surveys used the same response options as other media types. In the case of mobile phone use, MICS5 did not directly collect data on mother mobile phone usage. Instead, a question on whether any household member owned a mobile phone was used as a proxy to infer maternal access [48, 49]. While this proxy may not fully capture personal use, it serves as a practical indicator of access in resource-limited settings [50]. Differences in the design and timeframe between MICS5 and MICS6 were carefully addressed to ensure consistency in the analysis across the two waves.

In the literature, exposure to mass media is often measured in three ways: the use of individual media types, the number of media types used, and the frequency of media use [14, 16, 29]. This study applied all three approaches to comprehensively capture maternal media exposure.

To quantify media exposure, three methods were employed, these metrics allowed us to assess both the diversity and intensity of maternal media exposure.

1. Number of Media Types Used: A composite score ranging from 0 to 5, where 0 indicated no media use and 5 represented the use of all five media types (newspapers, radio, television, internet, and mobile phones) at least once a week or more frequently.
2. Frequency of Media Use: A composite score ranging from 0 to 15, based on the cumulative frequency of use across all media types. For example, a participant who reported “almost every day” for all five media types received a score of 15, while a participant who responded “not at all” to all media types scored 0.
3. Dichotomous Classification: For each media type, participants who reported using it at least once a week or more frequently were classified as users (coded as 1), while those who responded “not at all” were classified as non-users (coded as 0).

To account for potential confounding factors influencing early childhood development (ECD) outcomes, a comprehensive set of control variables was included. Child-specific characteristics such as age (categorized as 3 or 4 years), gender, and nutritional status (stunting, wasting, and underweight) were considered, as these directly affect development. Maternal and paternal characteristics included education levels, ages, and Parental engagement in stimulation activities was measured by whether parents participated in six activities with their children in the past three days: reading, storytelling, singing, outdoor activities, play, and naming, counting, or drawing were summarized into a continuous score ranging from 0 (no activities) to 6 (all activities). Household characteristics, such as size of household, wealth quintile, and the education level of the household head (used as a proxy for paternal education), were also incorporated. Additional covariates included the presence of children’s books in the household and salt iodization levels [26, 27], which served as a proxy for iodine intake critical to cognitive development. Regional factors such as urban or rural residence, provincial dummies, and district fixed effects were included to control for regional disparities in resources and infrastructure. These control variables were selected to ensure the observed effects of media exposure on ECD outcomes were not biased by demographic or socioeconomic differences, providing a robust and reliable analysis.

### Poisson model & its functional form

Descriptive statistics were used to compare rural and urban settings across exposure variables, outcome variables, and covariates, with chi-squared tests employed for categorical variables to examine group differences. Initially, logistic regression was used to explore the association between media use and early childhood development (ECD) across its four domains. However, given the high prevalence of ECD outcomes (over 65% of children were classified as ‘on track’), logistic regression produced inflated odds ratios, leading to an overestimation of the strength of associations. This is a common limitation of logistic regression for prevalent outcomes, as it tends to distort relative risks and yield misleading interpretations [51]. Recognizing this, we adopted Poisson regression with robust variance, which is widely recommended in epidemiological studies for modeling common outcomes. This method directly estimates prevalence ratios, avoiding the biases of odds ratios, and provides more reliable and interpretable results [52].

In Table 5, we present the results of five separate regressions examining the association between the types of media used by mothers and early childhood development (ECD) outcomes, as well as its four domains. Each regression corresponds to one of the ECD domains, allowing for a comprehensive assessment of the relationship between media use and child development. In Table 6, we explore a similar set of regressions, but this time focusing on the frequency of media use. Again, we run five regressions—one for ECD & rest for domains—to evaluate how varying levels of media exposure affect early childhood development. Finally, in Table 2, we regress each specific type of media—newspapers, television, radio, mobile phones, and the internet—on the overall ECD index and its four domains. This allows for a deeper understanding of how individual media types contribute to ECD outcomes. In all these models, relevant covariates and control variables were included to account for potential confounders and ensure that the relationships observed are robust and unbiased.

The functional form of the Poisson model is expressed as [52]:

$$\:\text{log}\left(E\left[{y}_{i}\right]\right)={{\upbeta\:}}_{0}+{{\upbeta\:}}_{1}{X}_{1i}+{{\upbeta\:}}_{2}{X}_{2i}+\dots\:+{{\upbeta\:}}_{p}{X}_{pi}$$

Where β_0​_ is the intercept & β_1+*i*_ are the regression coefficients for the predictor variables & $\:({X}_{1i},{X}_{2i},\dots\:,{X}_{pi})$ are predictor variables, such as media exposure (e.g., number or frequency of media use), maternal education, household wealth, and other covariates.

Incidence Rate Ratio (IRR) are derived from the model using the formula:

$$\:\text{I}\text{R}\text{R}={\text{e}}^{{{\upbeta}}_{\text{j}}}$$

This functional form illustrates how predictors, such as the composite score of media use or other covariates, are associated with the expected ECD outcomes. This approach ensured that the associations observed were robust and not inflated, providing a clear understanding of the relationship between media exposure and ECD outcomes across rural and urban populations.

### Hypothesis

To explore the relationship between media exposure and early childhood development (ECD), this study examines how different media types, their frequency of use, and variations across urban and rural areas influence ECD outcomes.

### Hypothesis 1: ECD and media type and frequency

Our hypothesis is that increased media exposure, measured through the number of media types and their frequency of use, is positively associated with ECD outcomes. Greater diversity and higher frequency of media usage by mothers are expected to enhance child development outcomes by providing parenting knowledge and access to health-related information.

### Hypothesis 2: ECD and individual media types

Individual media types, such as television, radio, newspapers, mobile phones, and the Internet, may have varying effects on ECD outcomes. We hypothesize that each media type contributes differently, with some media types (e.g., mobile phones) potentially having a stronger association due to their interactive and accessible nature.

### Hypothesis 3: ECD and area-specific differences in media use

We hypothesize that the relationship between media exposure (type and frequency) and ECD outcomes differs across urban and rural areas. Urban populations are expected to benefit more from media exposure due to better infrastructure, access to healthcare, and educational opportunities, while rural areas may experience reduced but still impactful effects of media use. Research highlights the vital role of media in enhancing ECD outcomes by equipping parents, especially mothers, with essential knowledge and resources, with urban populations benefiting more due to better access (Chen et al., 2018; Zahnd, 2009; Prado-Galbarro et al., 2021). Even in resource-limited rural areas, traditional media like TV and radio positively impact child development, while urban environments amplify these effects through broader media availability.

### Hypothesis 4: ECD and year-specific differences in media use

To explore year-specific differences in the relationship between media use and ECD, we included a year dummy variable and interacted it with media exposure variables (number and frequency of media types). This approach allowed us to assess how the impact of media use on ECD outcomes varied across the two survey waves.

## Results

Table presents socio-demographic characteristics and media usage patterns in relation to Early Childhood Development (ECD) outcomes across two groups: ECD on track and ECD not on track. In 2014, urban children had higher percentages of being on track (81.45%) compared to rural children (76.36%). However, by 2018, urban children’s ECD on track percentage decreased to 69.08%, while rural children showed a larger decline to 62.78%. Malnutrition also had a significant impact, with children who were malnourished showing lower ECD percentages, especially in 2018 (59.95% on track for malnourished children vs. 68.10% for those without malnutrition).

Media usage patterns reveal that children whose mothers used more media types generally had higher ECD percentages. For example, children whose mothers used four media types had 87.33% on track in 2014, which decreased to 68.60% in 2018. The mother’s education level was another key factor, with children of mothers who had higher education levels showing the highest percentage of ECD on track (89.51% in 2014). Wealth also played a significant role; children from the richest quintile had the highest ECD percentages, with 87.99% on track in 2014, but this decreased to 67.07% in 2018. Lastly, media use, especially mobile phones and the internet, was associated with better ECD outcomes, with children of mothers who used the internet showing 100% ECD on track in 2014.

**Table 2:** Weighted socio-demographic characteristics of children and use of five forms of media by mothers of children in two groups of ECD

Variables	Overall %	Year-2014		Year-2018	
		ECD on track %	ECD not on track %	ECD on track %	ECD not on track %
Residence					
Urban	27.65	81.45	18.55	69.08	32.27
Rural	72.35	76.36	23.64	62.78	37.22
Province					
Punjab	32.30	81.29	18.71	81.21	18.79
Sindh	19.11	66.57	33.43	65.51	34.49
Balochistan	18.09	-	-	57.96	42.04
KPK	26.61	82.53	17.47	68.80	31.20
Gilgit-Baltistan	3.90	77.67	22.33	-	-
Children’s age in years					
3	51.49	74.61	25.39	61.75	38.25
4	48.51	81.19	18.81	66.68	33.32
Children’s Gender					
Male	51.48	77.38	22.26	78.12	21.88
Female	48.52	64.13	35.87	64.21	35.79
Malnutrition^1^					
Yes	50.29	73.87	26.13	59.95	40.05
No	49.71	82.11	17.89	68.10	31.90
Child ever attended early childhood education programme					
Yes	84.09	90.74	9.26	60.38	39.62
No	15.91	75.22	24.78	85.44	14.56
Types of media					
0	16.84	63.89	36.11	47.00	53.00
1	38.04	75.57	24.43	65.98	34.02
2	33.85	79.44	20.56	72.28	72.28
3	8.89	84.30	15.70	22.03	77.97
4	2.23	87.33	12.67	68.60	31.40
5	0.15	56.08	43.92	80.90	19.10
Mother’s education level					
None/pre-school	64.39	73.67	26.33	59.07	40.93
Primary	12.59	80.49	19.51	74.39	25.61
Middle	6.50	84.11	15.89	72.27	27.73
Secondary	8.99	84.80	15.20	71.71	28.29
Higher	7.53	89.51	10.49	78.44	21.56
Wealth quintile of household					
Poorest	21.87	67.36	32.64	61.10	38.90
Poorer	20.59	74.31	25.69	65.35	34.65
Middle	19.96	79.47	20.53	62.96	37.04
Richer	18.97	82.09	17.91	64.78	35.22
Richest	18.62	87.99	12.01	67.07	32.93
Do you read newspapers or magazines?					
Yes	12.72	85.85	14.15	73.77	26.23
No	87.28	83.36	16.64	63.44	36.56
Frequency of listening to the radio					
Yes	5.46	79.56	20.44	64.94	35.06
No	94.54	77.62	22.38	64.16	35.84
Frequency of watching television					
Yes	44.75	80.20	19.80	71.53	28.47
No	55.25	75.46	24.54	58.76	41.24
Mobile phone usage in the past three months^2^					
Yes	79.27	78.60	21.40	70.78	29.22
No	20.73	64.86	35.14	48.93	51.07
Internet usage^3^					
Yes	82.82	100.00	0	81.21	18.79
No	17.18	85.14	14.86	61.26	38.74

^1^Children who were stunted or wasted or underweight

^2^In MICS-5 (2014), mobile phone ownership was not directly asked, so we use the household survey question, 'Does anyone in the household own a phone?' In 2018, mothers were directly asked about the frequency of their mobile phone use over the past three months

^3^In MICS-5 the question asks the frequency of internet usage in 1 month and for MICS-6 (2018) it refers to frequency of internet usage in 3 months

Table 4 highlights significant rural-urban differences in early childhood development (ECD) outcomes and socio-demographic factors. Urban children have a higher proportion (72.8%) on track for ECD compared to rural children (67.77%), with this difference being statistically significant (*p* < 0.01). Urban children in Punjab and Khyber Pakhtunkhwa (KPK) also show higher ECD rates (84.5% and 80%, respectively) compared to rural children in these regions, while no significant difference is observed in Balochistan. Urban children are more likely to have attained early childhood education (23.2% vs. 14.01%), and malnutrition rates are significantly higher in rural areas (52.71%) than urban areas (45.8%). Media exposure is also higher in urban areas, with urban mothers using 1.76 types of media per week compared to rural mothers at 1.12 types per week.

**Table 3:** Rural-urban comparison of study factor, outcome variable and other covariates

Variables (%)	Urban	Rural	*P*-value
Early childhood development on track	72.8	67.77	< 0.01
Early childhood development on track (by Province)			
Punjab	84.5	79.59	< 0.01
Sindh	69.6	59.08	< 0.01
Balochistan	41.4	43.33	0.08
KPK	80	71.14	< 0.01
Gilgit-Baltistan	79.5	76.91	0.28
Malnutrition^1^	45.8	52.71	< 0.01
Child ever attended early childhood education programme (ECE)	23.2	14.01	< 0.01
Children’s Gender (Male\Female)	51.87/49.16	50.83/48.12	0.012
Number of children ever born (Frequency)	3.8	4.2	< 0.01
Children’s average age (in years)	3.48	3.48	0.29
Children’s book present in house			
None	7.93	9.07	< 0.01
1–2 Books present	8.08	5.14	< 0.01
≥ 3 Books present	11.9	4.12	< 0.01
Average number of media types used less than once in a week or more^2^	1.85	1.24	< 0.01
Mother’s education level			
None/pre-school	43.3	71.73	< 0.01
Primary	14.4	12.26	< 0.01
Middle	9.48	5.18	< 0.01
Secondary	15.2	6.09	< 0.01
Higher	16.8	4.62	< 0.01
Average household size	2.46	2.57	< 0.01
Household head education level			
None/Preschool	35.5	53.24	< 0.01
Primary	14	14.6	0.013
Middle	11.7	9.78	< 0.01
Secondary	18.6	13.49	< 0.01
Higher	18.3	8.41	< 0.01
Wealth quintile of household			
Poorest	5.9	35.06	< 0.01
Poorer	1.81	26.81	< 0.01
Middle	19.4	19.48	0.79
Richer	28.7	12.48	< 0.01
Richest	35.7	6.15	< 0.01

^1^Children who were stunted or wasted or underweight

^2^For internet, the question refers to the last 1-month for 2014 & 3 months for 2018

### Number of media types used by mother & ECD

Table 5 column 1 demonstrates that a greater diversity in maternal media use is positively associated with early childhood development (ECD) outcomes. Specifically, each additional type of media used by the mother corresponds to a 5% increase in the likelihood of the child being developmentally on track in rural areas (aPR: 1.05; 95% CI: 1.04–1.06). This effect is amplified in urban areas, where the interaction term indicates an 8.15% increase (1.05 × 1.03), suggesting that media diversity may be even more impactful in contexts with better infrastructure and access to digital resources.

**Table 4:** Poisson regression with robust variance examining the association between the number of media types used by mother on early childhood development (ECD) & 4 domains

Variables	(ECD) (95% CI) 1	Four domains of ECD (95% CI)			
		(Literacy-numeracy domain) 2	(Physical domain) 3	(Learning domain) 4	(Social-emotional domain) 5
Number of media types used	1.05*** (1.04,1.06)	0.99 (0.96,1.03)	1.04*** (1.03,1.04)	1.05*** (1.04,1.05)	1.04*** (1.03,1.05)
Number of media types*area	1.03*** (1.01,1.04)	1.01 (0.99,1.04)	1.01** (1.00,1.02)	1.02** (1.01,1.02)	1.02** (1.01,1.03)
Media types*frequency of use	0.99*** (0.99,0.99)	1.00 (1.00,1.00)	0.99*** (0.99,0.99)	0.99*** (0.99,0.99)	0.99*** (0.99,0.99)
Types of media*year	1.07*** (1.05,1.08)	1.06*** (1.03,1.09)	1.07*** (1.06,1.07)	1.06*** (1.05,1.07)	1.04*** (1.03,1.06)
Area (Rural ref.)					
Urban	0.97* (0.94,0.99)	1.09** (1.02,1.16)	0.98 (0.97,1.00)	0.98* (0.96,1.00)	0.95*** (0.93,0.97)
Child’s age (3 years ref.)					
4 years	1.06*** (1.05,1.08)	1.54*** (1.50,1.58)	1.01** (1.00,1.01)	1.03*** (1.02,1.03)	1.01 (1.00,1.01)
Child’s gender (Female ref.)					
Male	1.01* (1.00,1.02)	0.99 (0.96,1.01)	1.00 (1.00,1.01)	1.00 (0.99,1.01)	1.02*** (1.01,1.03)
Malnutrition (No ref.)					
Yes	0.97*** (0.96,0.98)	0.81*** (0.79,0.83)	0.99** (0.99,1.00)	0.98*** (0.97,0.99)	0.98*** (0.97,0.99)
Early childhood education (No ref.)					
Yes	1.10*** (1.09,1.11)	2.13*** (2.07,2.20)	1.03*** (1.02,1.04)	1.02*** (1.01,1.02)	1.02** (1.01,1.03)
Childs book present in household (No ref.)					
1–2	1.07*** (1.05,1.08)	1.41*** (1.36,1.47)	1.01* (1.00,1.02)	1.02* (1.00,1.03)	1.02** (1.01,1.04)
≥ 3	1.06*** (1.04,1.07	1.31*** (1.26,1.35)	0.99 (0.98,1.00)	1.02*** (1.01,1.03)	1.03** (1.01,1.04)
Mother’s education (None/pre-school ref.)					
Primary	1.02** (1.00,1.03)	1.12*** (1.07,1.16)	1.00 (0.99,1.01)	1.01* (1.00,1.02)	1.00 (0.99,1.01)
Middle	1.00 (0.98,1.02)	1.12*** (1.07,1.16)	1.00 (0.99,1.01	0.99 (0.98,1.00)	0.99 (0.97,1.00)
Secondary	1.00 (0.98,1.01)	1.19*** (1.14,1.24)	1.00 (0.99,1.01)	0.99 (0.98,1.00)	0.97*** (0.95,0.99)
Higher	1.01 (0.99,1.02)	1.20*** (1.14,1.25)	0.99 (0.98,1.00)	0.98** (0.97,0.99)	0.99 (0.97,1.01)
Mother’s age (15–19 years ref.)					
20–24 years	1.09* (1.02,1.16)	1.15 (0.95,1.40)	1.04* (1.00,1.08)	1.03 (0.98,1.07)	1.10** (1.03,1.16)
25–29 years	1.08* (1.01,1.16)	1.19 (0.98,1.44)	1.04 (1.00,1.08)	1.02 (0.97,1.06)	1.08** (1.02,1.15)
30–34 years	1.07 (1.00,1.15)	1.19 (0.98,1.45)	1.03 (0.99,1.07)	1.01 (0.97,1.06)	1.08* (1.01,1.14)
35–39 years	1.08* (1.00,1.13)	1.21 (0.99,1.47)	1.04 (1.00,1.08)	1.01 (0.97,1.06)	1.08** (1.02,1.15)
40–44 years	1.05 (0.98,1.13)	1.20 (0.98,1.46)	1.02 (0.98,1.06)	1.00 (0.96,1.05)	1.06 (0.99,1.12)
45–49 years	1.04 (0.96,1.12)	1.20 (0.98,1.46)	1.02 (0.98,1.07)	1.00 (0.96,1.05)	1.03 (0.97,1.12)
Father’s age (15–24 years ref.)					
25–34 years	1.03 (1.00,1.06)	0.98 (0.90,1.06)	1.03*** (1.02,1.05)	1.03** (1.02,1.06)	1.04** (1.01,1.06)
35–44 years	1.04* (1.00,1.07)	1.00 (0.92,1.09)	1.03*** (1.02,1.05)	1.04*** (1.02,1.06)	1.04** (1.01,1.07)
45–54 years	1.04* (1.00,1.07)	1.06 (0.96,1.17)	1.03** (1.01,1.05)	1.04*** (1.02,1.07)	1.03* (1.00,1.06)
55 + years	1.08*** (1.03,1.12)	1.10 (0.98,1.23)	1.05*** (1.03,1.08)	1.05*** (1.02,1.08)	1.09*** (1.06,1.13)
Number of children born	1.01*** (1.00,1.01)	0.99** (0.98,1.00)	1.00*** (1.00,1.01)	1.01*** (1.00,1.01)	1.0*** (1.00,1.01)
Parental stimulation activities in the last 3-day (No ref.)					
Yes	1.02*** (1.00,1.02)	1.12*** (1.11,1.13)	1.00* (1.00,1.00)	1.00** (0.99,1.00)	1.01*** (1.00,1.00)
Household head education (None/pre-school ref.)					
Primary	1.01* (1.00,1.03)	1.04* (1.00,1.09)	1.01*** (1.01,1.02)	1.01 (1.00,1.02)	0.99 (0.98,1.01)
Middle	1.01 (0.99,1.02)	1.09*** (1.04,1.140	1.00 (0.99,1.01)	1.00 (0.99,1.01)	0.99 (0.98,1.01)
Secondary	1.02* (1.00,1.03)	1.13*** (1.09,1.17)	1.01 (1.00,1.01)	1.00 (0.99,1.01)	0.99 (0.98,1.01)
Higher	1.03** (1.01,1.04)	1.16*** (1.11,1.20)	1.01** (1.00,1.02)	1.01 (0.99,1.02)	0.99 (0.98,1.01)
Household size (Small households 2–4 ref.)					
Medium households (5–8)	1.00 (0.98,1.01)	0.99 (0.95,1.04)	0.99 (0.98,1.00)	0.99* (0.97,1.00)	1.01 (0.99,1.02)
Large households (9–12)	1.00 (0.99,1.02)	1.00 (0.99,1.01)	1.00 (0.99,1.01)	1.00 (0.99,1.01)	1.02* (1.00,1.04)
Very large households (13+)	1.05*** (1.03,1.07)	1.02 (0.97,1.07)	1.02*** (1.01,1.03)	1.02** (1.01,1.04)	1.05*** (1.03,1.07)
Wealth quintile index (Poorest ref.)					
Poorer	1.05*** (1.03,1.06)	1.40*** (1.33,1.48)	1.00 (0.99,1.01)	1.03*** (1.02,1.04)	1.01 (1.00,1.02)
Middle	1.04*** (1.02,1.05)	1.57*** (1.49,1.65)	0.98*** (0.97,0.99)	1.03*** (1.01,1.04)	0.99 (0.98,1.00)
Richer	1.03*** (1.01,1.05)	1.66*** (1.55,1.75)	0.97*** (0.95,0.98)	1.03*** (1.01,1.04)	0.97*** (0.96,0.99)
Richest	1.05*** (1.03,1.08)	1.65*** (1.55,1.75)	0.98*** (0.97,0.99)	1.06*** (1.05,1.08)	0.99 (0.97,1.01)
Salt iodization (Not iodized 0 PPM ref.)					
More than 0 PPM & less than 15 PPM	1.02* (1.00,1.03)	1.01 (0.98,1.05)	0.98*** (0.97,0.99)	1.03*** (1.02,1.03)	1.00 (0.99,1.01)
15 PPM or more	1.02*** (1.01,1.04)	1.03* (1.00,1.07)	0.99 (0.99,1.00)	1.03*** (1.02,1.03)	1.01 (1.00,1.02)
Year (2014 ref.)					
2018	0.90*** (0.88,0.92)	0.89*** (0.84,0.94)	0.86*** (0.85,0.87)	0.89*** (0.87,0.90)	0.97** (0.95,0.99)
Provinces (Punjab ref.)					
Sindh	0.55*** (0.52,0.58)	0.43*** (0.38,0.50)	0.77*** (0.75,0.79)	0.69*** (0.41,0.47)	0.72*** (0.41,0.49)
Balochistan	0.27*** (0.24,0.34)	0.18*** (0.14,0.24)	0.53*** (0.50,0.56)	0.44*** (0.41,0.47)	0.44*** (0.43,0.49)
Kpk	0.29*** (0.25,0.34)	0.12*** (0.08,0.19)	0.60*** (0.55,0.65)	0.44*** (0.39,0.48)	0.49*** (0.43,0.56)
Gilgit-Baltistan	0.20*** (0.17,0.25)	0.03*** (0.02,0.05)	0.52*** (0.46,0.58)	0.36*** (0.32,0.41)	0.40*** (0.34,0.48)
Districts FE	X	X	X	X	X
Observation	68,790	68,544	68,669	68,486	68,678

However, a significant but modest negative interaction between media type diversity and media use frequency (aPR: 0.99; 95% CI: 0.99–0.99) suggests that while diverse media exposure is beneficial, excessive or high-frequency use across multiple platforms might slightly hinder developmental outcomes. This could reflect overstimulation or reduced caregiver-child interaction, which prior research has identified as potential risks of screen overuse. Notably, the interaction with survey year shows an enhanced effect in 2018 (12.35%), indicating a growing influence of media over time—possibly due to the increasing penetration of smartphones and digital content.

Four-year-old children show a higher ECD score than three-year-old (1.06, 95% CI: 1.05–1.08), indicating developmental progress with age. Boys slightly outperform girls (1.01, 95% CI: 1.00-1.02). Malnutrition is associated with a reduction in ECD scores by 3%, reinforcing its adverse impact on development. Early childhood education (1.10, 95% CI: 1.09–1.11) and book availability (1.07 for 1–2 books, 1.06 for ≥ 3 books) improve ECD. Maternal primary education (aPR: 1.02; 95% CI: 1.00–1.03), maternal age (20-44) [11, 13, 42], and paternal age (particularly 55+) all show positive effects, possibly reflecting greater parenting knowledge, experience, or household stability. Additional positive associations were found with wealth quintiles, parental stimulation, iodized salt use, education of the household head, and larger household size (aPR: 1.05; 95% CI: 1.03–1.07). Interestingly, compared to Punjab, all other provinces display significantly lower ECD scores.

### Number of media types used by mother & 4 domains of ECD

The column (2-5) of Table 5 explore the relationship between maternal media use and the four domains of Early Childhood Development (ECD): Literacy-Numeracy, Physical, Learning, and Social-Emotional. The number of media types used by mothers is significantly and positively associated with all four domains, with the strongest effects observed in the Physical, Learning, and Social-Emotional domains (aPR range: 1.04–1.05). These findings suggest that exposure to diverse media may enrich the caregiving environment and support a broad range of developmental skills beyond cognitive gains alone.

The interaction between media use and residential area reveals nuanced effects. While rural children benefit from increased media exposure, the gains are comparatively modest in the Physical and Learning domains. In contrast, urban children exhibit more pronounced improvements in the Social-Emotional domain, possibly due to better access to quality media content or more supportive home environments that amplify the impact of media.

Beyond media-related factors, several socio-demographic characteristics are significantly associated with ECD outcomes. Urban residence is linked to higher Literacy-Numeracy scores, underscoring disparities in access to learning resources. Children aged 4 years have higher scores in all domains, particularly in the Literacy-Numeracy domain, with a 54% increase in ECD scores. Malnutrition continues to show a detrimental impact, especially in the Literacy-Numeracy and Learning domains, highlighting the interdependence of health and cognitive development. Participation in early childhood education emerges as a robust predictor of improved scores across all domains, especially in Literacy-Numeracy. Additionally, higher maternal education levels and household wealth are consistently associated with better ECD outcomes, with particularly strong effects in the Literacy-Numeracy and Physical domains. These findings emphasize the importance of both structural and proximal factors—such as education, nutrition, and household resources—in shaping early development.

### Frequency of media & ECD

In Table 6 column 6 we discuss the frequency by which the mother used media and its impact on ECD. For each additional unit of media frequency use, the prevalence of child being on track for ECD increased by 3% (aPR: 1.03; CI: 1.02–1.03) for rural area. This suggests that more frequent exposure to media is linked with a higher probability of children being on track. The interaction term between frequency of media use and area indicated that the association was slightly stronger in urban areas, where the prevalence increased by 4% (1.03*1.01). The interaction between the number of media types and the frequency of use (0.99, 95% CI: 0.99–0.99) reveals a small but significant negative relationship. This suggests that although exposure to different types of media is beneficial for ECD, using them excessively across multiple platforms might slightly impede developmental progress. Additionally, the interaction between media types and the year shows a 4% increase in the effect for 2018 (1.03*1.01), indicating a slight boost in the impact of media on ECD outcomes compared to previous years.

Boys have slightly higher ECD scores compared to girls, while malnutrition negatively impacts ECD outcomes. Early childhood education, having books in the household, and maternal education are all positively associated with better ECD scores. Father’s age, parental stimulation, and the educational level of the household head also show positive correlations with ECD development.

### Frequency of media use by mother & 4 domains of ECD

In column (7-8-9-10) of Table 6 shows the analysis highlights significant factors influencing early childhood development (ECD) across four domains. Media exposure, particularly the frequency of media use, and early childhood education have strong positive associations with ECD outcomes. Child’s gender, malnutrition, household education levels, and parental stimulation activities also play notable roles in enhancing development. Regional differences, with provinces like Punjab showing higher ECD scores, further emphasize contextual factors in shaping developmental outcomes. Lastly, household wealth and the presence of books in the home notably support child development.

**Table 5:** Poisson regression with robust variance examining the associations between media types and early childhood development

Variables	(ECD) (95% CI) 6	Four domains of ECD (95% CI)			
		(Literacy-numeracy domain) 7	(Physical domain) 8	(Learning domain) 9	(Social-emotional domain) 10
Frequency of media use	1.03*** (1.02,1.04)	1.02* (1.00,1.04)	1.02*** (1.02,1.02)	1.03*** (1.02,1.03)	1.02*** (1.01,1.03)
Frequency of media use*area	1.01*** (1.01,1.02)	1.02*** (1.01,1.03)	1.00** (1.01,1.02)	1.01*** (1.00,1.01)	1.01*** (1.00,1.01)
frequency of use* Media types	0.99*** (0.99,0.99)	0.99*** (0.99,1.00)	0.99*** (0.99,1.00)	0.99*** (0.99,0.99)	0.99* (0.99,1.00)
Frequency of media use*year	1.01*** (1.01,1.02)	1.01* (1.00,1.03)	1.01*** (1.01,1.02)	1.01*** (1.01,1.02)	1.01*** (1.01,1.01)
Area (Rural ref.)					
Urban	0.97** (0.95,0.99)	1.02 (0.97,1.07)	0.99 (0.98,1.00)	0.99 (0.97,1.00)	0.95*** (0.94,0.97)
Child’s age (3 years ref.)					
4 years	1.06*** (1.05,1.08)	1.54*** (1.50,1.58)	1.01** (1.00,1.01)	1.03*** (1.02,1.03)	1.01 (1.00,1.01)
Child’s gender (Female ref.)					
Male	1.01* (1.00,1.02)	0.99 (0.96,1.01)	1.00 (1.00,1.01)	1.00 (0.99,1.01)	1.02*** (1.01,1.03)
Malnutrition (No ref.)					
Yes	0.97*** (0.96,0.98)	0.81*** (0.79,0.83)	0.99** (0.99,1.00)	0.98*** (0.97,0.99)	0.98*** (0.97,0.99)
Early childhood education (No ref.)					
Yes	1.10*** (1.09,1.11)	2.13*** (2.07,2.20)	1.03*** (1.02,1.04)	1.02*** (1.01,1.02)	1.02** (1.01,1.03)
Childs book present in household (No ref.)					
1–2	1.07*** (1.05,1.09)	1.41*** (1.36,1.47)	1.01* (1.00,1.02)	1.02* (1.00,1.03)	1.02** (1.01,1.04)
≥ 3	1.06*** (1.04,1.08)	1.31*** (1.26,1.35)	0.99 (0.98,1.00)	1.03*** (1.01,1.04)	1.03** (1.01,1.05)
Mother’s education (None/pre-school ref.)					
Primary	1.02** (1.01,1.04)	1.11*** (1.07,1.16)	1.00 (0.99,1.01)	1.01* (1.00,1.02)	1.00 (0.99,1.01)
Middle	1.00 (0.98,1.02)	1.19*** (1.14,1.25)	1.00 (0.99,1.01	0.99 (0.98,1.00)	0.99 (0.97,1.00)
Secondary	1.00 (0.98,1.01)	1.19*** (1.14,1.24)	1.00 (0.99,1.01)	0.99 (0.98,1.00)	0.97*** (0.95,0.99)
Higher	1.01 (0.99,1.03)	1.20*** (1.14,1.25)	0.99 (0.98,1.00)	0.98** (0.97,0.99)	0.99 (0.97,1.01)
Mother’s age (15–19 years ref.)					
20–24 years	1.09* (1.02,1.17)	1.16 (0.95,1.40)	1.04* (1.00,1.09)	1.03 (0.98,1.08)	1.10** (1.04,1.17)
25–29 years	1.08* (1.01,1.16)	1.19 (0.98,1.45)	1.04* (1.00,1.08)	1.02 (0.97,1.06)	1.09** (1.02,1.15)
30–34 years	1.07* (1.00,1.15)	1.19 (0.98,1.45)	1.03 (0.99,1.08)	1.01 (0.97,1.06)	1.08* (1.01,1.14)
35–39 years	1.08* (1.01,1.16)	1.21 (0.99,1.47)	1.04 (1.00,1.08)	1.01 (0.97,1.06)	1.08** (1.02,1.15)
40–44 years	1.05 (0.98,1.13)	1.20 (0.98,1.47)	1.02 (0.98,1.07)	1.00 (0.96,1.05)	1.06 (0.99,1.12)
45–49 years	1.04 (0.96,1.12)	1.23 (0.98,1.46)	1.02 (0.98,1.07)	1.00 (0.95,1.05)	1.03 (0.97,1.12)
Father’s age (15–24 years ref.)					
25–34 years	1.02 (0.99,1.05)	0.98 (0.90,1.06)	1.03*** (1.01,1.05)	1.03** (1.01,1.05)	1.03** (1.01,1.06)
35–44 years	1.03* (1.00,1.07)	1.00 (0.91,1.09)	1.03*** (1.01,1.05)	1.03*** (1.01,1.06)	1.04** (1.01,1.06)
45–54 years	1.03 (1.03,1.12)	1.06 (0.96,1.17)	1.03* (1.01,1.05)	1.04** (1.01,1.06)	1.03 (1.00,1.06)
55 + years	1.07*** (1.03,1.12)	1.09 (0.97,1.23)	1.05*** (1.03,1.08)	1.05** (1.02,1.08)	1.09*** (1.05,1.13)
Number of children born	1.01*** (1.00,1.01)	0.99** (0.98,1.00)	1.00*** (1.00,1.01)	1.01*** (1.00,1.01)	1.00*** (1.00,1.01)
Parental stimulation activities in the last 3-day (No ref.)					
Yes	1.02*** (1.01,1.02)	1.12*** (1.11,1.13)	1.00* (1.00,1.00)	1.00** (0.99,1.00)	1.01*** (1.00,1.00)
Household head education (None/pre-school ref.)					
Primary	1.02* (1.00,1.03)	1.04* (1.00,1.09)	1.02*** (1.01,1.02)	1.01 (1.00,1.02)	1.00 (0.98,1.01)
Middle	1.01 (0.99,1.03)	1.09*** (1.04,1.140	1.00 (0.99,1.01)	1.00 (0.99,1.01)	0.99 (0.98,1.01)
Secondary	1.02* (1.00,1.03)	1.13*** (1.09,1.17)	1.01 (1.00,1.01)	1.00 (0.99,1.01)	0.99 (0.98,1.01)
Higher	1.03*** (1.01,1.05)	1.15*** (1.11,1.20)	1.02** (1.00,1.03)	1.01 (0.99,1.02)	1.00 (0.98,1.01)
Household size (Small households 2–4 ref.)					
Medium households (5–8)	1.00 (0.98,1.02)	1.00 (0.95,1.04)	0.99 (0.98,1.00)	0.99 (0.98,1.00)	1.01 (0.99,1.02)
Large households (9–12)	1.01 (0.99,1.03)	1.01 (0.99,1.07)	1.00 (0.99,1.01)	1.00 (0.99,1.01)	1.02* (1.00,1.04)
Very large households (13+)	1.05*** (1.03,1.07)	1.02 (0.97,1.08)	1.03*** (1.01,1.04)	1.03*** (1.01,1.04)	1.05*** (1.04,1.07)
Wealth quintile index (Poorest ref.)					
Poorer	1.05*** (1.03,1.06)	1.40*** (1.33,1.48)	1.00 (0.99,1.01)	1.02*** (1.01,1.03)	1.01 (1.00,1.02)
Middle	1.04*** (1.02,1.06)	1.57*** (1.49,1.65)	0.98*** (0.97,0.99)	1.02*** (1.01,1.04)	0.99 (0.98,1.00)
Richer	1.03* (1.01,1.05)	1.66*** (1.55,1.75)	0.97*** (0.95,0.98)	1.03*** (1.01,1.04)	0.97*** (0.96,0.99)
Richest	1.05*** (1.03,1.07)	1.63*** (1.53,1.73)	0.98*** (0.97,0.99)	1.05*** (1.04,1.07)	0.99 (0.97,1.01)
Salt iodization (Not iodized 0 PPM ref.)					
More than 0 PPM & less than 15 PPM	1.02* (1.00,1.03)	1.01 (0.98,1.05)	0.98*** (0.97,0.99)	1.03*** (1.02,1.03)	1.00 (0.99,1.01)
15 PPM or more	1.02*** (1.01,1.04)	1.03* (1.00,1.07)	0.99 (0.99,1.00)	1.02*** (1.02,1.03)	1.01 (1.00,1.02)
Year (2014 ref.)					
2018	0.90*** (0.88,0.91)	0.90*** (0.86,0.94)	0.86*** (0.86,0.87)	0.88*** (0.87,0.90)	0.97** (0.95,0.99)
Provinces (Punjab ref.)					
Sindh	0.54*** (0.52,0.57)	0.43*** (0.37,0.49)	0.76*** (0.74,0.79)	0.69*** (0.66,0.71)	0.71*** (0.68,0.75)
Balochistan	0.26*** (0.23,0.29)	0.18*** (0.14,0.24)	0.52*** (0.48,0.55)	0.42*** (0.40,0.46)	0.43*** (0.43,0.47)
Kpk	0.28*** (0.24,0.33)	0.12*** (0.08,0.18)	0.58*** (0.55,0.65)	0.42*** (0.38,0.47)	0.48*** (0.42,0.55)
Gilgit-Baltistan	0.19*** (0.16,0.23)	0.03*** (0.02,0.05)	0.50*** (0.45,0.56)	0.35*** (0.30,0.39)	0.38*** (0.32,0.46)
Districts FE	X	X	X	X	X
Observation		68,544	68,669	68,486	68,678

### Types of media used by mother & ECD

The table presents the association of various factors with early childhood development (ECD). Notably, mobile phone use by mothers is strongly linked to ECD outcomes for rural areas, for urban there is no impact (interaction term between the mobile and area). While other media types such as newspapers, radio, television, and the internet show no impact. Urban areas, male children, early childhood education, and parental stimulation activities positively affect ECD. Regional differences are significant, with provinces like Balochistan and KPK exhibiting lower ECD outcomes. Factors like household size, parental education, and salt iodization also influence ECD, though wealth quintile and year have less of an impact.

### Types of media used by mother & 4 domains of ECD

The findings across the four domains of Early Childhood Development (ECD)—literacy-numeracy, physical, learning, and social-emotional—highlight several key patterns. Mobile media usage shows the most substantial and statistically significant positive impact on all domains. Early childhood education strongly enhances development, particularly in the literacy-numeracy and learning domains. The presence of books in households positively contributes to development, with three or more books having a greater effect. Child age positively influences the literacy-numeracy domain for four-year-old, while boys exhibit higher scores in the social-emotional domain. Malnutrition negatively impacts learning development, whereas parental stimulation positively influences all domains. Urban children tend to have better outcomes in the literacy-numeracy domain compared to rural counterparts. Mother’s and household head’s education also show varying degrees of positive association with development outcomes. Household size has a moderate impact, with larger households linked to better physical development. Wealth status shows minimal influence across the domains, while iodized salt consumption presents limited association. Lastly, regional disparities persist, with Punjab outperforming other provinces, and Balochistan significantly lagging in the physical domain.

**Table 6:** Poisson regression with robust variance examining the associations between media types and early childhood development

Variables	(ECD) (95% CI) 11	Four domains of ECD (95% CI)			
		(Literacy-numeracy domain) 12	(Physical domain) 13	(Learning domain) 14	(Social-emotional domain) 15
Types of media used by mother					
Newspaper	1.03 (0.98,1.10)	1.03 (0.89,1.18)	1.02 (0.99,1.06)	0.99 (0.95,1.03)	1.06 (1.00,1.14)
Newspaper*area	0.95 (0.89,1.03)	0.90 (0.76,1.07)	0.99 (0.95,1.03)	1.00 (0.95,1.05)	0.93 (0.86,1.02)
Radio	0.98 (0.88,1.09)	1.09 (0.89,1.33)	1.03 (0.97,1.10)	0.99 (0.92,1.06)	0.98 (0.88,1.09)
Radio*area	0.99 (0.86,1.13)	0.93 (0.71,1.21)	0.92 (0.84,1.00)	0.98 (0.89,1.08)	1.01 (0.87,1.17)
Television	1.04 (0.96,1.13)	1.08 (0.90,1.28)	1.02 (0.97,1.07)	1.04 (0.98,1.09)	1.02 (0.94,1.10)
Television*area	0.95 (0.85,1.07)	0.88 (0.68,1.12)	0.97 (0.90,1.05)	0.94 (0.87,1.01)	1.01 (0.94,1.10)
Internet	1.04 (0.95,1.14)	1.17 (0.95,1.44)	1.00 (0.95,1.06)	1.00 (0.94,1.05)	1.06 (0.96,1.16)
Internet*area	0.99 (0.88,1.12)	0.94 (0.72,1.23)	1.06 (0.97,1.15)	1.03 (0.96,1.11)	0.98 (0.86,1.12)
Mobile	1.93*** (1.40,2.65)	1.49 (0.87,2.54)	1.46*** (1.20,1.77)	1.69*** (1.35,2.12)	1.47*** (1.14,1.89)
Mobile*area	0.71 (0.44,1.14)	0.71 (0.34,1.49)	0.81 (0.59,1.10)	0.78 (0.56,1.08)	0.85 (0.57,1.26)
Area Rural (ref.)					
Urban	1.50 (0.94,2.41)	1.85 (0.91,3.80)	1.23 (0.90,1.68)	1.35 (0.97,1.88)	1.21 (0.82,1.78)
Child’s age 3 years (ref.)					
4 years	1.02 (0.98,1.06)	1.58*** (1.44,1.74)	0.99 (0.96,1.02)	0.98 (0.95,1.01)	0.95* (0.91,0.99)
Child’s gender Female (ref.)					
Male	1.04* (1.00,1.08)	0.97 (0.89,1.05)	1.01 (0.99,1.04)	1.01 (0.99,1.04)	1.04* (1.00,1.09)
Malnutrition No (ref.)					
Yes	0.98 (0.94,1.03)	0.93 (0.84,1.02)	0.99 (0.96,1.02)	0.96** (0.93,0.99)	0.98 (0.93,1.03)
Early childhood education No (ref.)					
Yes	1.09*** (1.05,1.14)	1.59*** (1.45,1.74)	1.02 (1.00,1.05)	1.05*** (1.02,1.08)	1.06* (1.01,1.11)
Childs book present in household No (ref.)					
1–2	1.03 (0.98,1.09)	1.19** (1.06,1.33)	1.02 (0.99,1.06)	1.04* (1.00,1.07)	0.99 (0.93,1.05)
≥ 3	1.01 (0.96,1.06)	1.25*** (1.14,1.37)	0.99 (0.96,1.03)	1.03 (1.00,1.06)	0.94* (0.88,1.00)
Mother’s education None/pre-school (ref.)					
Primary	1.06 (0.93,1.20)	1.10 (0.83,1.45)	1.02 (0.94,1.10)	1.07 (0.99,1.16)	1.07 (0.95,1.20)
Middle	1.01 (0.89,1.15)	1.20 (0.90,1.58)	0.97 (0.89,1.06)	1.02 (0.94,1.11)	1.02 (0.89,1.15)
Secondary	1.07 (0.95,1.20)	1.14 (0.88,1.47)	1.01 (0.94,1.14)	1.06 (0.99,1.14)	1.11 (0.89,1.15)
Higher	1.06 (0.95,1.19)	1.09 (0.84,1.41)	1.00 (0.92,1.08)	1.07 (1.00,1.15)	1.07 (0.96,1.20)
Mother’s age 15–19 years (ref.)					
20–24 years	1.03 (0.74,1.43)	0.73 (0.40,1.36)	0.93 (0.77,1.12)	1.01 (0.80,1.27)	1.53 (0.92,2.53)
25–29 years	1.06 (0.76,1.47)	0.75 (0.40,1.38)	0.94 (0.78,1.14)	1.04 (0.82,1.32)	1.52 (0.92,2.52)
30–34 years	1.06 (0.76,1.48)	0.73 (0.40,1.36)	0.92 (0.76,1.11)	1.03 (0.82,1.31)	1.58 (0.96,2.62)
35–39 years	1.09 (0.78,1.53)	0.76 (0.41,1.42)	0.95 (0.78,1.15)	1.03 (0.81,1.30)	1.65 (1.00,2.75)
40–44 years	1.02 (0.72,1.44)	0.87 (0.46,1.65)	0.95 (0.77,1.16)	1.07 (0.84,1.36)	1.51 (0.90,2.54)
45–49 years	0.95 (0.60,1.51)	0.74 (0.31,1.73)	0.92 (0.68,1.24)	0.92 (0.67,1.26)	1.71 (0.96,3.04)
Father’s age 15–24 years (ref.)					
25–34 years	0.96 (0.83,1.10)	1.54 (0.93,2.56)	1.08 (0.96,1.22)	1.02 (0.91,1.14)	0.93 (0.81,1.08)
35–44 years	0.98 (0.84,1.13)	1.61 (0.96,2.69)	1.08 (0.96,1.22)	1.01 (0.91,1.13)	0.92 (0.79,1.07)
45–54 years	0.94 (0.79,1.11)	1.60 (0.93,2.76)	1.03 (0.90,1.19)	1.01 (0.90,1.15)	0.82* (0.68,0.99)
55 + years	1.20 (0.94,1.52)	1.56 (0.84,2.89)	1.28** (1.07,1.55)	1.03 (0.84,1.28)	1.02 (0.75,1.39)
Number of children born	0.99 (0.97,1.01)	0.94** (0.90,0.98)	0.99 (0.97,1.00)	1.01 (0.99,1.00)	1.02*** (1.01,1.04)
Parental stimulation activities in the last 3-day No (ref.)					
Yes	1.03*** (1.01,1.04)	1.09*** (1.06,1.11)	1.01** (1.01,1.02)	1.00 (0.99,1.00)	1.02*** (1.01,1.04)
Household head education None/preschool (ref.)					
Primary	1.04 (0.96,1.12)	1.16 (0.99,1.37)	1.00 (0.95,1.05)	0.99 (0.95,1.05)	0.99 (0.91,1.07)
Middle	1.06 (0.98,1.14)	1.09 (0.92,1.28)	1.02 (0.98,1.07)	1.04 (1.00,1.09)	1.00 (0.92,1.08)
Secondary	1.07* (1.00,1.13)	1.19** (1.04,1.36)	1.01 (0.97,1.05)	1.02 (0.98,1.06)	1.02 (0.96,1.09)
Higher	1.07* (1.01,1.14)	1.20** (1.05,1.37)	1.02 (0.98,1.06)	1.01 (0.97,1.05)	1.00 (0.93,1.07)
Household size small households (2–4) (ref.)					
Medium households (5–8)	1.00 (0.95,1.06)	1.00 (0.89,1.13)	1.03 (1.00,1.07)	0.96* (0.93,1.00)	0.97 (0.91,1.04)
Large households (9–12)	1.04 (0.98,1.11)	0.97 (0.84,1.11)	1.07** (1.02,1.11)	0.99 (0.95,1.03)	1.01 (0.94,1.08)
Very large households (13+)	1.11** (1.03,1.19)	0.93 (0.79,1.09)	1.07* (1.02,1.12)	1.01 (0.96,1.06)	1.01 (0.94,1.08)
Wealth quintile index Poorest (ref.)					
Poorer	0.97 (0.72,1.30)	1.79 (0.86,3.75)	0.97 (0.81,1.17)	0.89 (0.72,1.09)	1.03 (0.80,1.35)
Middle	0.95 (0.72,1.26)	1.72 (0.83,3.54)	0.89 (0.75,1.06)	0.89 (0.72,1.08)	1.03 (0.81,1.32)
Richer	0.93 (0.71,1.23)	1.49 (0.73,3.08)	0.88 (0.74,1.05)	0.93 (0.77,1.12)	0.97 (0.76,1.24)
Richest	0.99 (0.75,1.30)	1.87 (0.91,3.84)	0.90 (0.76,1.07)	0.94 (0.78,1.13)	0.97 (0.76,1.25)
Salt iodization Not iodized 0 PPM (ref.)					
More than 0 PPM & less than 15 PPM	0.97 (0.90,1.04)	1.02 (0.88,1.19)	0.96 (0.92,1.19)	1.00 (0.95,1.05)	1.00 (0.93,1.09)
15 PPM or more	0.97 (0.91,1.04)	1.03 (0.89,1.18)	0.98 (0.93,1.02)	0.98 (0.94,1.03)	0.99 (0.92,1.07)
Year 2014 (ref.)					
2018	0.99 (0.90,1.10)	0.84 (0.67,1.05)	0.98 (0.94,1.03)	0.94* (0.89,1.00)	1.09 (0.96,1.25)
Provinces Punjab (ref.)					
Sindh	0.71** (0.55,0.92)	0.84 (0.49,1.44)	0.83* (0.71,0.96)	0.84* (0.71,0.99)	0.90 (0.68,1.18)
Balochistan	0.36*** (0.21,0.61)	0.41 (0.13,1.27)	0.51*** (0.37,0.70)	0.72 (0.51,1.03)	0.62 (0.35,1.10)
Kpk	0.41* (0.19,0.88)	0.41 (0.08,2.19)	0.65 (0.41,1.04)	0.75 (0.45,1.25)	0.73 (0.32,1.68)
Gilgit-Baltistan	0.36* (0.13,1.00)	0.25 (0.03,2.28)	0.59 (0.32,1.10)	0.74 (0.37,1.45)	0.71 (0.23,2.21)
Districts FE	X	X	X	X	X
Observation	2493	2489	2492	2486	2492

## Conclusion

This study reveals the multifaceted role of maternal media engagement in shaping early childhood development (ECD) outcomes in Pakistan, with distinct patterns across rural and urban settings. Exposure to diverse media types, particularly mobile phones, is positively linked to developmental progress, especially in rural areas where mobile use doubles the likelihood of children meeting age-appropriate milestones. In urban settings, the cumulative effect of diverse media exposure is even stronger, with each additional media type associated with over an 8% improvement in ECD outcomes. However, the findings also suggest that while media diversity is generally beneficial, excessive frequency of media use across multiple platforms may slightly hinder developmental progress, highlighting the need for balanced and purposeful media engagement. The temporal increase in media’s impact, notably a 12% rise by 2018, reflects the growing accessibility and influence of digital content on early learning environments. Domain-specific analyses show media’s strongest associations with physical and social-emotional development, whereas urban children perform better in literacy-numeracy. In addition to media-related factors, maternal education, early childhood education enrolment, availability of books in the home, and adequate nutrition significantly contribute to improved developmental outcomes. Conversely, malnutrition continues to be a major impediment to ECD, particularly in learning domains, while persistent provincial disparities—such as Punjab’s consistent outperformance—point to structural inequities in program reach and resource allocation.

Based on these findings, targeted and evidence-based policy measures are essential. For rural areas, mobile-based interventions offering curated content on parenting, health, and early learning can be a cost-effective strategy to enhance ECD outcomes. Simultaneously, promoting guidelines to mitigate media overexposure is critical to preserving the benefits of media engagement. Expanding equitable access to quality early childhood education—particularly in underserved provinces like Balochistan and KPK—should be integrated with nutrition programs and community-based growth monitoring to tackle malnutrition. Regional disparities require localized solutions, including investments in caregiver training, infrastructure development, and culturally tailored awareness campaigns. In urban contexts, strengthening programs that support literacy-numeracy and social-emotional skills through enriched environments and active parental involvement will further advance developmental outcomes. Finally, integrating media-driven behaviour change initiatives with broader efforts in maternal education and poverty alleviation can help reduce socioeconomic gaps and promote inclusive early childhood development across the country.

## Data Availability

This research uses data from Multiple Indicator Cluster Survey (MICS) data from wave 5 and 6 for Pakistan which is available at MICS website.Link to data; URL: https://mics.unicef.org/surveys?display=card&f[0]=region:4146&f[1]=datatype:0.

## Appendix

**Table 7:** Summary of key reviewed literature

Ref.	Study Focus	Key Findings	Gaps Identified	Relevance to Current Study
[10]	Explores how characteristics of the urban environment influence early childhood development (ECD) in Mexican children aged 36–59 months.	Finds that the availability of libraries and daycare centers is positively associated with better socio-emotional and literacy-numeracy development. Inadequate ECD was linked to high population density and marginalization.	The study mainly focused on urban settings, with limited comparison to rural contexts. It doesn’t address the influence of media in child development.	Offers insights into the impact of urban infrastructure on ECD, which may help contextualize the role of media and environmental factors in development outcomes.
[15]	Systematic review of 111 mass media campaigns targeting child survival health behaviors in low- and middle-income countries.	Moderate and stronger evaluations indicate positive impacts on behaviors such as immunization, oral rehydration therapy use, and early initiation of breastfeeding.	Limited information on campaign design elements, exposure levels, and theoretical frameworks.	Provides evidence of mass media’s effectiveness in influencing child survival behaviors, relevant to exploring media’s role in early childhood development outcomes.
[20]	Investigated the impact of mass media exposure on maternal healthcare utilization in India, Bangladesh, Nepal, and Pakistan using 2014–2017 Demographic and Health Surveys.	Women exposed to mass media were significantly more likely to receive antenatal care (46–86%), deliver with skilled birth attendants (24–53%), and receive postpartum check-ups (36–94%). The association was moderated by maternal education in three of the four countries.	Limited exploration of the mechanisms through which media exposure influences healthcare utilization. The study did not examine the role of specific media content or the frequency of exposure.	Provides evidence of mass media’s potential to improve maternal healthcare utilization, which is pertinent to exploring media’s role in early childhood development outcomes.
[22]	Systematic review across six health topics to assess effectiveness of mass media campaigns in changing health behaviors.	Moderate evidence of effectiveness for reducing sedentary behavior and improving sexual health behaviors. Mixed results for physical activity and tobacco use. Stronger effects noted in youth for tobacco/illicit drug campaigns. Campaign intensity, duration, and message targeting improved outcomes.	Limited evidence for diet, alcohol, and illicit drugs. Lacked statistical synthesis due to heterogeneity. Minimal focus on new media or theoretical frameworks.	Supports the broader role of mass media in health behavior change, providing transferable insights for media’s potential influence on parenting practices and child development, especially in resource-limited settings.
[26]	Examined changes in early childhood development (ECD) status and associated factors using two rounds of the Multiple Indicator Cluster Survey (MICS) in Bangladesh.	ECD outcomes improved over time: from 65.5% on-track in 2012 to 74.9% in 2019. Higher odds of being developmentally on-track were associated with: age (4 years vs. 3), being female, maternal education (secondary or higher), family wealth, and presence of books in the home. All four ECD domains improved across both survey years.	Does not explore the role of media or parenting practices as influencing factors. Also limited to a Bangladeshi context.	Supports the role of maternal education and family environment in ECD outcomes, which aligns with the current study’s focus. Adds longitudinal perspective on how socioeconomic factors shape ECD.
[29]	Examined the association between mothers’ use of five types of mass media and their children’s early childhood development (ECD) in urban and rural Bangladesh.	Found a positive association between the number of media types used by mothers and the likelihood of their children being on track in ECD. This association was stronger in rural areas (7% increase per additional media type) compared to urban areas (4% increase). Specific media types like newspapers, television, and internet were significantly associated with better ECD outcomes in rural areas, while radio was significant in urban areas.	The study is specific to the Bangladeshi context and may not be directly generalizable to other low- and middle-income countries.	Provides evidence supporting the role of mass media in enhancing ECD, especially in rural settings. This underscores the potential of leveraging various media platforms in similar contexts, such as Pakistan, to improve child development outcomes in rural context.

## Footnotes

1 The data is available at https://mics.unicef.org/surveys?display=card&f[0]=region:4146&f[1]=datatype:0
